# Pediatric Gunshot Head Injury: Prevalence of Prognostic Factors in Surgical Patients: An Institutional Experience in Ten Years

**DOI:** 10.1089/neur.2021.0024

**Published:** 2021-12-30

**Authors:** Luiz Severo Bem Junior, Otávio da Cunha Ferreira Neto, Artêmio José Araruna Dias, Pedro Lukas Do Rêgo Aquino, José Renan Miranda Cavalcante Filho, Andrey Maia Silva Diniz, Luís Felipe Gonçalves de Lima, Nilson Batista Lemos, Joaquim Fechine de Alencar Neto, Thais Lima Da Silva, Taciana Andrade De Abreu, João Guilherme De Lima Guerra Barros, Edvaldo Jeronimo da Silva Junior, Ana Cristina Veiga Silva, Igor Vilela Faquini, Nivaldo Sena Almeida, Hildo Rocha Cirne de Azevedo Filho

**Affiliations:** ^1^ Departament of Neurosurgery, Hospital da Restauração, Recife, Brazil.; ^2^ College of Medical Sciences, Unifacisa University Center, Campina Grande, Paraíba, Recife, Brazil.; ^3^ Catholic University of Pernambuco, Recife, Brazil.; ^4^ Oswaldo Cruz University Hospital, University of Pernambuco, Recife, Brazil.; ^5^ Federal University of Ceara, Sobral, Brazil.; ^6^ College of Medical Sciences, Federal University of Paraíba, João Pessoa, Brazil.; ^7^ Regional Hospital of Presidente Prudente, Sao Paulo, Brazil.

**Keywords:** craniocerebral trauma, gunshot wound, pediatrics

## Abstract

This article aims to evaluate the predictive factors of morbidity and mortality in pediatric patients who suffered gunshot wounds to the head. We reviewed a series of 43 patients who were admitted to a referential neurosurgical hospital between 2010 and 2019. Data from 43 patients who underwent a surgical treatment in our institution were collected, and the following parameters were considered in the analysis: the initial Glasgow Coma Scale (GCS), age, sex, bullet entry site, and bullet trajectory. Computed tomography (CT) scans at admission, complications, midline crossing, and Glasgow score scale at the time of discharge (Glasgow Outcome Scale; GOS) were also factored in. Male sex corresponded to 90.7% of cases (*N* = 39), and 16–17 years of age was the most common age (60.5%). The frontal region was the most common entry site (41.9%), followed by the parietal wall and occipital entry. Penetrating trajectory was shown in 48.8% of cases, perforation/transfixing in 39.5%, and tangential in 11.6%. CT showed that sinking is the most common alteration (74.4%), followed by cerebral contusion (44.2%). According to the GOS, 23.3% died, 23.3% were classified by an unfavorable outcome (GOS, 2–3), and 53.5% a favorable outcome (GOS, 4 and 5). In our study, there was a significant association between the low GCS scores on admission and low GOS (1–3; *p* = 0.001) at time of discharge. Patients with wounds that crossed the midline also had a significant association with low GOS (*p* = 0.014) in our clinical experience. We concluded that low GCS scores at admission and children with a wound that crosses the midline are predictive factors of high mortality and morbidity, in our clinical experience.

## Introduction

Gunshot wounds to the head are the most lethal of all penetrating brain injuries, and >90% of persons experiencing gunshot injuries to the head eventually die.^1,2^ The magnitude, management, and neurological deficits with penetrating head injuries are well established in the literature.^3^ Harvey Cushing was a pioneer in the management of penetrating head injuries, and he popularized the craniectomy, debridement, and closure for treating penetrating brain injuries. The method was replaced by osteoplastic craniotomy by Cairns in World War II.^1,4^

Penetrating traumatic brain injuries (TBIs) are common in war or conflict; however, TBIs in a civil population are uncommon.^5,6^ In the larger cities of Brazil, the prevalence of urban violence is high, because of the conflicts of drugs gangs in peripheral communities, in addition to armed conflicts with the police. Domestic violence or accidents are sometimes responsible for inciting larger numbers in child mortality by gunshot, as well as examples of child-related actions. The human development index for the city of Recife is 0.772 (WHO, 2015); however, there are big disparities in the poorest neighborhoods with a lack of social security and little medical assistance.^7^ In Recife, the death rate for homicides in adolescents is higher than the death rate for transport accidents, having been shown in a study, with a 78.8% homicide death rate.^8^

Victims vary from the children to adult populations; the incidence of gunshot injuries to the head in pediatric cases is rare and therefore poorly reported in the literature. In general, a patient admitted with a low score in the Glasgow Coma Scale (GCS) or damage to the cerebral ventricles will have a bad prognosis.^5,6^

This article aims to identify the prevalence of prognostic factors in pediatric surgical patients with gunshot wounds to the head over the past 10 years, at our neurosurgery department. All patients included in this study were submitted to surgical treatment.

## Methods

This is a retrospective study in which all pediatric patients were submitted to surgical procedures because of the perforation by firearms to the skull at the Hospital da Restauração (Recife, Brazil) during the period of January 2010 to December 2019, an institutional experience of 10 years. Previous university institutional review board approval had been obtained.

**FIG. 1.**
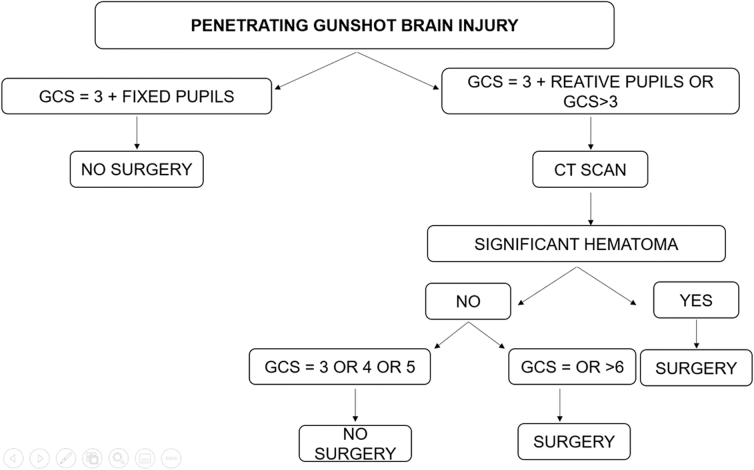
Flowchart depicting penetrating gunshot brain injury. CT, computed tomography; GCS, Glasgow Coma Scale.

To reduce the patient's heterogeneity and allow an adequate analysis to take place, our inclusion criterion for this study was to include only surgical patients according to the institution's flow (flowchart; Fig. 1) and patients <18 years of age, according to the World Health Organization classification for pediatric patients. Therefore, our exclusion criteria were those ∼19 years of age, non-surgical patients, and patients without complete information for analysis.

A large craniotomy aimed at the exposure of the damaged dura and brain tissue, including the entrance wound, was accomplished. Surgical treatment included: resection of necrotic tissue; drainage of hematomas; vigorous cleaning of the foreign bodies and bone fragments; and the appropriate repair of dura, bone, and soft tissues. The following parameters were considered in the data analysis: the initial GCS, age, sex, bullet entry site, bullet trajectory, computed tomography (CT) scan findings, complications, midline crossing, and Glasgow score scale at discharge (Glasgow Outcome Scale; GOS).

Multi-variate analysis was used to compare two previously established groups based on the Glasgow admission. Patients were divided into the two groups according to their GCS score: group A (score from 9 to 15) and group B (scores from 3 to 8). By the time of hospital discharge, patients were classified into three groups according to the GOS: deaths (GOS, 1), unfavorable outcome (GOS, 2–3), and favorable outcome (GOS, 4–5).

### Statistical analysis

A multi-variate analysis was performed and a chi-square test was utilized to verify the correlation between the presence of the variables and the occurrence of an increased mortality rate and of an unfavorable outcome (GOS, 2 and 3). The margin of error used in the analysis of the statistical tests was 5%. The data were entered into the Excel worksheet (Microsoft Corporation, Redmond, CA), and the program used to obtain the statistical calculations was IBM SPSS software (version 23; SPSS, Inc., Chicago, IL).

## Results

### Patients' profile

The series of 43 pediatric patients with the diagnosis of gunshot wound to the head was comprised of 39 males (90.7%) and 4 females (9.3%) with a mean age of 15.44 years (range, 8–18). The highest incidence (60.5%) was in the second decade group showing data regarding the profile of patients participating in the study. All this information is summarized in Table 1.

**Table 1.** Evaluation of the Study Patients' Profile

Variant	Total group
Total, *n* (%)	**43 (100,0)**
Age: Mean ± SD (median)	15.44 ± 2.15 (16.00)
Age group (years), *n* (%)	
8–15	14 (32.5)
16–17	26 (60.5)
18	3 (7.0)
Sex, *n* (%)	
Male	39 (90.7)
Female	4 (9.3)

SD, standard deviation.

### Clinical data

The frontal region was the most frequent bullet entry site, with 44.4% of the group, followed by the parietal (30.6%) and occipital (22.2%) regions. Faces and temporal sites were recorded in 11.1% and 8.3%, respectively. The highest percentage (47.2%) had a penetrating trajectory, followed by perforating/transfixing (38.9%) and tangential trajectory (13.9%).

The most frequent diagnoses of CT were: sinking skull (77.8%), cerebral contusion (38.9%), and cerebral hemorrhage (36.1%).

Complications were presented in 11 patients (27.9%). Infection on surgical site and cerebrospinal fluid fistula occurred in 8 patients (18.6%), no epidural or abscess occurred, motor deficit in 3 patients (7.0%), and epilepsy in 1 patient (2.3%).

In 25% of patients, the bullet crossed the midline, in 36.1% the bullet did not cross, and in 38.9% this information was not described.

According to the GCS, 21 patients (48.8%) were classified into group A and 22 patients (51.1%) into group B. According to the GOS, 23.3% were classified by an unfavorable outcome (GOS, 2–3) and 53.5% a favorable outcome (GOS, 4 and 5). The mortality in our study was 23.3%. Table 2 summarizes all this information.

**Table 2.** Evaluation of Clinical Data

Variant	Total group
Total, *n* (%)	**43 (100.0)**
Bullet entry site, *n* (%)^a^	
Frontal	18 (41.9)
Parietal	12 (27.9)
Occipital	9 (20.9)
Face	4 (9.3)
Temporal	6 (14.0)
Bullet trajectory, *n* (%)	
Penetrating	21 (48.8)
Perforating/transfixing	17 (39.5)
Tangential	5 (11.6)
CT scan: n (%)^a^	
Sinking skull	32 (74.4)
Brain contusion	19 (44.2)
Subarachnoid hemorrhage	15 (34.9)
Subdural hematoma	2 (4.7)
Intraparenchimal hematoma	4 (9.3)
Unknown	3 (7.0)
Complications, *n* (%)^a^	
Infection	
Motor Impairment	8 (18.6)
Epilepsy	3 (7.0)
None	1 (2.3)
	32 (74.4)
Midline crossing, *n* (%)	
Yes	9 (20.9)
No	20 (46.5)
Unknown	14 (32.6)
Glasgow Coma Scale (GCS) on admission, *n* (%)	
3–8	21 (48.8)
9–15	22 (51.1)
Glasgow Outcome Scale (GOS) at discharge, *n* (%)	
Death	10 (23.3)
Unfavorable/vegetative	10 (23.3)
Favorable	23 (53.5)

^a^ Considering that the same patient could have been affected by more than one situation, the basis for calculating the percentages, not the total, is recorded.

CT, computed tomography.

Patients were categorized into the following two age groups: 8–15 and >16. Of the 14 patients in the 8–15 age group, 3 (21.4%) died and 4 (40%) had an unfavorable outcome. In the group of patients >16 years, the mortality rate was 24.1% (7 patients) and the unfavorable outcome rate was 60% (6 patients; Table 3).

**Table 3.** GOS Analyze According to Age Group, Bullet Trajectory, Computed Tomography, Crossing with the Midline and GCS at Admission

			GOS at discharge (outcome)							
	Death		Unfavorable (1–3)		Favorable (3–5)		Total			
Variant	n	%	n	%	n	%	N	%	p value	OR (95% CI)
Age									*p*^c^ = 0.750	
8–15	3	21.4	4	40	7	50	14	100		1.23 (0.34–4.42)
>16	7	24.1	6	60	16	55.2	29	100		1
**Total group**	**10**	**23.2**	**10**	**23.2**	**23**	**53.5**	**43**	**100**		
Bullet trajectory									*p*^d^ = 0.522	
Penetrating	10	47.6	0	0	11	52.4	21	100		b
Perforating/transfixing	2	11.7	7	41.1	8	47.1	17	100		b
Tangential	1	20	0	0	4	80	5	100		b
**Total group**	**13**	**32.5**	**20**	**46.5**	**23**	**53.5**	**43**	**100**		
CT scan										
Sinking skull									*p*^d^ = 1.000	
Yes	7	21.8	7	21.8	18	56.3	32	100		1
No	1	12.5	3	37.5	4	50	8	100		1.29 (0.27–6.07)
**Total group**	**8**	**20**	**9**	**22.5**	**22**	**55**	**40**	**100**		
Cerebral contusion									*p*^c^ = 0.726	
Yes	3	15.7	5	29.3	11	57.9	19	100		1
No	3	14.3	7	33.3	11	52.4	21	100		1.25 (0.36–4.36)
**Total group**	**6**	**15**	**12**	**30**	**22**	**55**	**40**	**100**		
Cerebral hemorrhage									*p*^c^ = 0.622	
Yes	1	6,6	5	33.3	9	60	15	100		1
No	3	12	9	36	13	52	25	100		1.38 (0.38–5.07)
**Total group**	**4**	**10**	**14**	**35**	**22**	**55**	**40**	**100**		
Midline crossing									*p*^d^ = 0.014^a^	
Yes	3	33.3	4	44.4	2	22.2	9	100		10,50 (1.62–68.07)
No	3	15	2	10	15	75	20	100		1
**Total group**	**6**	**20.6**	**6**	**20.6**	**17**	**58.6**	**29**	**100**		
GCS at admission									*p*^c^ = 0.001^a^	
Group A (3–8)	6	28.5	9	42.8	6	28.6	21	100		8.50 (2.15–33.62)
Group B (9–15)	4	18.2	1	4.5	17	77.3	22	100		1
**Total group**	**10**	**23.2**	**10**	**23.2**	**23**	**53.5**	**43**	**100**		

^a^ Significant association at the 5.0% level.

^b^ Could not be determined because of the occurrence of very low frequencies.

^c^ Through Pearson's chi-square test.

^d^ Using Fisher's exact test.

GOS, Glasgow Outcome Scale; GCS, Glasgow Coma Scale; OR, odds ratio; 95% CI, 95% confidence interval.

Incidence of death in the group with penetrating, perforating/transfixing, and tangential bullet trajectory was 47.6%, 11.7%, and 20%, respectively. In analyzing the CT scan findings, incidence of death in the group with sinking skull, cerebral contusion, and cerebral hemorrhage was 21.8%, 15.7%, and 6.6%, respectively. Thus, age, bullet trajectory, and CT scan findings had no significant association (*p* > 0.05) with GOS at the time of discharge (Table 3).

Low GSC at the time of admission and wound midline crossing had a significant association (*p* < 0.05, odds ratio [OR] = 6.72, and a range that excludes the value 1.00) with the GOS at the time of discharge and a bad prognosis. Of the 21 cases from group A, 6 patients (28.5%) died and 9 patients (42.8%) had an unfavorable outcome. Of 9 cases in which there was a midline crossing, 3 patients (33.3%) died and 4 patients (44.4%) had an unfavorable outcome. Thus, low GCS at admission and a midline crossing wound are predictive factors of high mortality and morbidity in pediatric patients with gunshot wounds to the head after being submitted to surgical treatment (Table 3).

There was no statistically significant relationship between the most frequent CT findings and the trajectory of the bullet (sinking skull, *p* = 0.285; cerebral contusion, *p* = 0.549; cerebral hemorrhage, *p* = 0.810). This analysis is shown in Table 3.

## Discussion

Gunshot injury is the most lethal of the penetrating brain injuries, with reported lethal rates from 85% to 93%.^7,8^ In our study, the mortality rate in the pediatric population who suffered gunshot-based head injuries and who underwent a surgical treatment was 23.3%. The pathophysiological mechanisms in gunshot injuries involve energy translation and tissue cavitation and can cause respiratory and cardiac arrest. The exact pathophysiology mechanism of apnea in a penetrating brain injury is not clean.^9^ There are several hypotheses. Anatomical studies have demonstrated that a direct function of the energy deposited by the missile may affect the respiratory neurons of the medullary respiratory center. Another hypothesis showed that the low cardiac output leading to a decrease in cerebral perfusion causes the damage in respiratory neurons and apnea.^10^ The principal pathological effects of the craniocerebral system are brain swelling, intracranial hemorrhage, and penetrating injury with bone and metal fragments and other foreign bodies.^11^

Ballistic aspects of the wounding should always be considered, including the type of weapon used, the proximity at the time of discharge, bullet caliber, jacketing, and velocity.^12^ The volume of injured brain and size of cavitation adjacent to the path of the missile are dependent on the kinetic energy imparted to the brain by the missile.^12^ This depends on the velocity of the missile at the point of impact with the head and the thickness of the skull. The extent of the brain injury also depends on the size, shape, spin, and yaw of the missile and whether or not it fragments.^12^

The score of the GCS to indicate a surgical procedure in penetrating TBI is still controversial, and some researchers have different opinions. In a prospective study in 1990, Grahm and colleagues did not recommended surgery for patients with a GCS score of 3–5. However, immediate surgery in this study was recommended for patients with a GCS of >8 and for patients with an operable hematoma.^13^ In another study, Cavaliere and colleagues^14^ recommended surgery on those patients with a GCS of >6, even though one half of these patients died post-operatively.

Some researchers recommend or advise higher aggressiveness on debridement.^9^ However, the timing of surgery in gunshot injuries is important because early surgery may also decrease post-operative complications.^15^ Minor pellet injuries to the brain with small entry wounds may only require local debridement, closure, and antibiotics. More severe focal injuries with hemorrhage and fragments without adverse radiological features may also only require local exploration through a small craniotomy.^15,16^ More severe penetrating injuries will require extensive surgery and may include decompressive craniectomy, debridement, evacuation of hematomas, dural repair, and insertion of an intracranial pressure monitor.^12^ Antibiotics and anticonvulsant drugs are recommended in post-operative treatments.^12^ Infectious complications are not uncommon after penetrating TBI, and they are also associated with poor prognosis, with cephalosporins being the most preferred antibiotic.^16^

In our study, 18.6% of patients developed infection and all of those used antibiotic regimen for prophylaxis. In our protocol, patients with admissions of GCS 3 without reflexes were not indicated for surgery. Admission GCS of between 3 and 6 were indicated minimal debridement local and above 6, and we were aggressive. Prognostic factors in patients with craniocerebral firearm projectile wounds have been studied broadly, mainly through retrospective studies within a civilian population. There are several clinical findings and imaging features that are significant determinants of outcomes.^12,17^ These include age, admission GCS, abnormal pupil reactivity, and the trajectory of the missile and obliteration of the basal cisterns. Other factors have been considered important for the prognosis as well, including: respiratory and hemodynamic; the missile type; the pupil diameter and its reactivity; and CT findings.^12,18,19^ The presence of subarachnoid hemorrhage (SAH) in penetrating TBI has been documented to correlate significantly with mortality and morbidity.^12^ The frequency of SAH in penetrating TBI patients ranges from 31% to 78% in the literature (including pediatric and adult patients).^12^ In our study, the frequency of SAH was 39.4% in the pediatric population and had no significant association with the outcome.

There is no consensus in the literature regarding the age of the patient as a prognostic factor to penetrating gunshot injuries of the head.^12,18,19^ In our study, age was not a significant association with the GOS at discharge. The mode of injury, such as penetrating, tangential, and perforating, was associated with a worse outcome.^12^ However, in our study, with pediatric patients this variable had no significant association with the outcome. CT scan findings also had no association with low GOS at the time of discharge in our experience.

In accordance with other researchers,^12–14,17^ the presence of low GCS at the time of admission was a significant association with high morbidity and mortality in our study. Low GCS may indicate edema- or hematoma-induced compression of brainstem structures maintaining consciousness, or direct damage to these structures by the wound tract.^20^ In pediatric patients with a wound that crossed the midline, there was a significant association with low GOS at discharge and bad prognosis.

Firearm projectile head wounds have become a frequent neurosurgical emergency in many cities in Brazil. There are multiple clinical, radiological, and surgical factors that may influence the prognosis of a patient upon admission into a hospital's emergency room. We conclude that low GCS scores at admission and wound midline crossing had a significant association with GOS at the time of discharge. Thus, these variables are predictive factors of high mortality and morbidity in children with gunshot wounds to the head submitted to surgical treatment, in our clinical experience.

### Limitation of the study

The limitations of our study are directly associated with retrospective data analysis, only being in one neurosurgical center and with a low frequency of the trauma being discussed, containing a relatively small number of patients.

## Notes

**Cite this article as:** Bem Junior, LS, Ferreira Neto, OC, Araruna Dias, AJ, Do Rêgo Aquino, PL, Miranda Cavalcante Filho, JR, Silva Diniz, AM, Gonçalves de Lima, LF, Lemos, NB, de Alencar Neto, JF, Lima Da Silva, T, Andrade De Abreu, T, De Lima Guerra Barros, JG, da Silva Junior, EJ, Veiga Silva, AC, Faquini, IV, Almeida, NS, Rocha Cirne de Azevedo Filho, H (2021) Pediatric gunshot head injury: prevalence of prognostic factors in surgical patients: an institutional experience in ten years. *Neurotrauma Reports* 2:1, 669–675, DOI:10.1089/neur.2021.0024.

## Abbreviations Used

CT: computed tomography
GCS: Glasgow Coma Scale
GOS: Glasgow Outcome Scale
SAH: subarachnoid hemorrhage
TBIs: traumatic brain injuries
